# Acute Neurotoxicity in Children Treated for Acute Lymphoblastic Leukemia and Lymphoblastic Lymphoma: A 10-Year Single-Centre Experience

**DOI:** 10.3390/children12010031

**Published:** 2024-12-28

**Authors:** Izabela Kranjčec, Nada Rajačić, Tamara Janjić, Monika Kukuruzović, Filip Jadrijević-Cvrlje, Maja Pavlović, Jelena Roganović

**Affiliations:** 1 Department of Oncology and Hematology, Children’s Hospital Zagreb, Klaićeva 16, 10000 Zagreb, Croatia; izabela.kranjcec@kdb.hr (I.K.); filip.jadrijevic@kdb.hr (F.J.-C.); maja.pavlovic@kdb.hr (M.P.); jelena.roganovic@kdb.hr (J.R.); 2 General Hospital Karlovac, 47000 Karlovac, Croatia; t_janjic@hotmail.com; 3 Division of Neurology, Department of Pediatrics, University Hospital Centre ‘Sestre Milosrdnice’, EpiCARE, Vinogradska Cesta 29, 10000 Zagreb, Croatia; monika.kukuruzovic@kbcsm.hr; 4 Faculty of Biotechnology and Drug Development, University of Rijeka, 51000 Rijeka, Croatia

**Keywords:** leukemia, acute lymphoblastic, central nervous system, neurologic symptoms, antineoplastic agents

## Abstract

**Background**: Recent advances in childhood acute lymphoblastic leukemia (ALL) and lymphoblastic lymphoma (LL) management provide higher survival rates at the cost of increased toxicities. Acute neurotoxicity affects up to 10% of patients, requiring rapid recognition and treatment. **Methods**: A retrospective observational study was performed to determine the frequency, clinical manifestations, radiological characteristics, treatment options and outcome of acute neurological adverse events in pediatric patients with lymphoid malignancies at the Department of Oncology and Hematology, Children’s Hospital Zagreb, Croatia. **Results**: A total of 56 patients (48 ALL and 8 LL, male/female ratio 1:1, average age 5.4 years) were treated mainly according to the ALL-IC BFM 2009 protocol. The B-immunophenotype was the most frequent (85.7%). Most patients were stratified to the intermediate risk group (39.3%), and two were initially diagnosed with central nervous system infiltration. Acute neurotoxic events were registered in 11 patients (19.6%), most commonly in the 6–10-year age group (66.7%), predominately in females (72.7%) and high-risk group (54.5%). The most frequent clinical presentation was seizures (83.3%), with status epilepticus in four cases. We detected electroencephalogram (EEG) irregularities in almost all patients and various morphological changes in the brain magnetic resonance imaging (MRI), most often consistent with posterior reversible encephalopathy syndrome and leukoencephalopathy. Approximately half the patients received prolonged antiepileptic therapy. No apparent residual neurologic manifestations have been observed. **Conclusions:** Acute neurotoxicity is a rather frequent treatment-related adverse event, associated with high-risk disease. Early recognition and timely management are essential for rapid recovery and optimal outcomes.

## 1. Introduction

The successful treatment of acute lymphoblastic leukemia (ALL) and lymphoblastic lymphoma (LL) in children using modern chemotherapy protocols has led to an overall survival rate of over 90% in developed countries [1,2]. However, patients treated for hematological malignancies may face various adverse events, including neurological complications, which are disease- and treatment-related [3,4,5].

Complications involving the central nervous system (CNS) in patients with ALL are classified as primary and secondary. Primary complications are due to leukemic infiltration, while secondary complications arise from intensive and prolonged treatment [6,7,8,9].

Leukemic infiltration of the CNS is often asymptomatic and is typically detected only when lymphoblasts are found in the cerebrospinal fluid (CSF). When symptoms do occur, they may include seizures, loss of consciousness and signs associated with increased intracranial pressure, such as headache, vomiting, lethargy and papilledema [10].

CNS hemorrhage can also emerge, resulting from leukostasis, thrombocytopenia, and coagulopathy, the latter more commonly associated with acute myeloid leukemia [7].

Additional clinical manifestations are cranial nerve palsies, with the facial nerve being the most frequently affected [11,12], and signs arising from spinal cord compression by leukemic infiltrate [10].

Treatment-related, i.e., secondary CNS side effects include white matter lesions, small vessel calcifications, cerebrovascular disorders, secondary tumors and infections.

Neurologic complications of chemotherapy could present as cerebrovascular accidents, posterior reversible encephalopathy syndrome, subacute leukoencephalopathy, acute encephalopathy, syndrome of inappropriate antidiuretic hormone secretion and aseptic meningitis [13,14,15].

Neurotoxicity can also be classified as acute, subacute or chronic. Acute neurotoxicity is often transient with full recovery but requires rapid recognition and treatment.

In contrast, subacute and chronic neurotoxicity can be progressive, potentially leading to permanent consequences [16]. Long-term exposure to chemotherapy can lead to chronic CNS damage, such as chronic encephalopathy, chronic cognitive impairment and myelopathy. Clinical presentation varies according to the causative agent, degree of severity and time of onset [17].

Clinical manifestations of neurotoxicity differ significantly and often include disorders of consciousness, focal deficits, seizures [18,19], headaches and loss of vision and sensation. Limb weakness or numbness [20], memory loss and cognitive [21,22] and behavioral problems have also been reported [23,24].

Although acute neurotoxicity is transient, prompt recognition and management are crucial for a swift recovery and optimal therapeutic outcomes [25].

Given the significant clinical implications of acute neurological adverse events, this paper aims to present the experience related to the occurrence, clinical manifestations, diagnostic procedures, therapeutic approaches and outcomes of neurological adverse events during ALL and LL treatment in a tertiary pediatric oncology centre.

## 2. Materials and Methods

A retrospective observational study was conducted to evaluate acute central neurological complications in children treated for ALL and LL at the Children’s Hospital Zagreb, Croatia. The study included patients aged 0–17 years who were newly diagnosed with ALL and LL between 1 January 2012, and 31 December 2021.

The analysis focused on acute neurological adverse events that had a sudden onset and required immediate clinical attention and further investigations.

Central neurological adverse events, referred to in this study as neurotoxicity, are neurological manifestations—excluding psychiatric symptoms—that originate in the brain rather than from peripheral nerves, such as peripheral polyneuropathy.

Demographic data and disease characteristics (immunophenotype, CNS involvement, risk group) were retrieved from the patient’s electronic medical records. Children were treated according to the Berlin-Frankfurt-Münster (BFM) protocols (ALL-IC BFM 2009 Protocol in 54 patients and Interfant-06 Protocol in 2 patients as the first-line treatment).

The ALL IC BFM 2009 Protocol includes the induction phase (Protocol IA and Protocol IB), consolidation, re-induction (Protocol II) and maintenance therapy. Methotrexate (MTX), cytosine-arabinoside (Ara-C) and asparaginase are cytostatic drugs with possible side effects in the CNS and are components of the Protocol. MTX and Ara-C are administered intravenously (iv) and intrathecally, the latter always in combination with MTX and prednisolone for intrathecal administration. Systemically, MTX is administered at 2 or 5 g/m^2^ iv in the consolidation phase, while the child’s age determines the intrathecal dosage. During the maintenance phase, MTX is taken orally, weekly. The total number of intrathecal therapies depends on the initial CNS status. A single dose of iv Ara-C ranges from 75 mg/m^2^ to 2 g/m^2^.

In the presence of the initial leukemic CNS infiltration (CNS status 3), the neurocranium and C1-C2 vertebrae are irradiated at a therapeutic dose of 12 or 18 Gy, depending on age, upon completion of re-induction. Prophylactic CNS irradiation is no longer recommended due to observed late toxicity.

Clinical presentation and duration of the acute central neurotoxic events were recorded and graded according to the Common Terminology Criteria for Adverse Events (CTCAE) v. 5.0. In addition, we gathered information on diagnostic procedures like an electroencephalogram (EEG), computed tomography (CT) and magnetic resonance imaging (MRI), both at the time of diagnosis and cerebral events. Concomitant disorders (e.g., febrile neutropenia, electrolyte disturbance) at the time of the neurologic incident were registered. Antiepileptic therapy, if initiated, was noted, as was the information on neurologic follow-up and outcome.

The statistical analysis was performed using descriptive statistics in Microsoft Excel, and Fisher’s exact test was applied to evaluate gender, age and risk groups in GraphPad Prism 8.4.3. A significance level of *p* < 0.05 was adopted.

The study adhered to ethical standards and received approval from the institutional Ethical Committee on 29 November 2023 (Approval No. 01-23/37-4-23).

## 3. Results

### 3.1. Population Characteristics

During the 10 years, 56 patients (28 male) were treated for ALL and LL at our department, of whom 8 (14.3%) were diagnosed with LL. The average age at diagnosis was 5.4 years (median 4.3 years, range 0.5–15 years). Most (60.7%) of the patients were in the 1–5-year age group, while only two (3.6%) were treated in infancy.

Apart from the two infants treated according to the Interfant-06 Protocol, all other patients were treated according to the ALL-IC BFM 2009 protocol as a first-line therapy. One patient experienced late medullary relapse and was treated according to the ALL-REZ BFM Protocol as a second-line treatment, followed by an allogeneic stem cell transplant.

The predominant immunophenotype (85.7%) was of B-cell origin. Twenty-five percent of patients were stratified in a standard-risk group, 39.3% in an intermediate-risk group and 21.4% in a high-risk group. Two children underwent cranial radiotherapy (CRT) due to CNS involvement (CNS 3).

All patients are alive and remain in complete remission, with a median follow-up of 6.5 years (mean of 7.6 years, range 5–12 years).

### 3.2. Acute Neurotoxic Events

Acute neurotoxic events were registered in 11 patients (19.6%). One patient experienced late medullary relapse, and adverse central neurological events in the first- and second-line treatment were documented, with a total of 12 events in our cohort being analysed.

Central neurological complications were most common in the age group 6–10 years (8/12 events; 66.7%) and predominated in the female gender (8/11; 72.7%). Neither of the infants (one male and one female) manifested central neurotoxicity.

Of 11 patients with central neurotoxicity, only one had T-immunophenotype ALL/LL (9.1%), with no indication for cranial radiotherapy. All B-immunophenotype intermediate-risk patients received MTX 2 g/m^2^ iv during the consolidation phase of the treatment, except one who received 5 g/m^2^ and experienced an adverse central neurological event.

All high-risk patients received MTX 5 g/m^2^. One out of two children who received CRT developed acute central neurotoxicity.

The majority of patients with central neurotoxicity were stratified into a high-risk group (54.5%), with three into an intermediate-risk group (27.3%) and two into a standard-risk group (18.2%).

None of the patients with LL had acute neurological toxicity, although they received the same chemotherapy as patients with ALL.

Out of 12 events, 4 (33.3%) were recorded during the induction phase (2 in Protocol IA and 2 in Protocol IB), 6 (50%) in the re-induction (5 in ALL-IC BFM 2009 Protocol II and 1 in ALL-REZ BFM Protocol II-IDA) and 1 (8.3%) during consolidation, i.e., in high-risk block 1 (HR-1 block). One event (8.3%) occurred during the maintenance therapy (8.3%). Nine intrathecal MTX therapies were applied within 21 days of the event (mean 6.7 days, median 6.0 days, range 2–17 days). Five native asparaginase applications occurred within 21 days of the event (mean 7.2 days, median 8.0 days, range 2–12 days). Only asparaginase in the pegylated form has been used at our centre since 2021.

Fisher’s exact test failed to demonstrate a statistically significant difference among groups regarding the age of six years and older (*p* = 0.0567) and sex (*p* = 0.1771), while the high-risk group was significantly related to central neurotoxic events (*p* = 0.0176).

### 3.3. Clinical Manifestations

The most common clinical manifestations were seizures (83.3%). One (8.3%) event was described as a reduced level of consciousness, and one (8.3%) as headache and vomiting without other manifestations. All the seizures were generalised, mostly tonic-clonic (40%) and tonic (30%). One attack was atonic (10%), and two were complex partial seizures with secondary generalisation (20%). The seizures lasted from 30 s to 2 h. Four attacks progressed to status epilepticus (40%), three of which required endotracheal intubation.

The clinical presentation of all 12 neurologic events is described in Table 1.

According to CTCAE v. 5, the adverse event was most often of moderate severity (grade III; 58.3%), while the least common was a mild event (grade II; 16.7%). Three incidents were described and graded as severe and life-threatening (grade IV; 25%).

Four neurologic events occurred during febrile neutropenia, with sterile blood cultures in four and sterile CSF in one event. In all events, arterial hypertension necessitating antihypertensive therapy was registered, including both posterior reversible encephalopathy syndrome (PRES) and cerebrovascular ischemia. Two cases of concomitant electrolyte disbalances were observed, one registered in a patient with PRES. The patient with PRES had hyponatremia, hypokalemia, hypocalcemia, hypophosphatemia and hypomagnesemia needing correction.

### 3.4. Diagnostics

#### 3.4.1. EEG

At the time of diagnosis, nearly half of the patients (45.5%) did not undergo an EEG.

The same percentage of patients had a normal initial EEG result. One EEG was irregular but did not demonstrate any asymmetry or specific graphoelements. A brain CT scan was performed in seven patients (63.6%), and the results were unremarkable.

Additionally, brain MRI was not part of the initial routine work-up.

EEG recordings were conducted 11 times in 10 patients during adverse neurological events, with abnormal patterns found in nine recordings (Table 1). An EEG was omitted only in one patient presenting with headache and vomiting.

#### 3.4.2. Imaging

At the time of diagnosis, neuroimaging was not performed in nearly one-third of the patients (36.4%), and the only imaging technique used was a brain CT scan.

Following the acute neurotoxic event, eleven brain MRIs and one brain CT scan were conducted. The most common MRI finding was white matter changes associated with leukoencephalopathy (LE), observed in five patients. This occurred after six adverse events, as one patient experienced two incidents. PRES was identified in two MRIs, while one MRI confirmed cerebrovascular ischemia in the territory of the left posterior cerebral artery. Additionally, two MRIs showed no significant abnormalities. In two patients, neuroimaging revealed multiple conditions: one patient had LE accompanied by a pontine cavernoma and mineralising microangiopathy following radiotherapy, while the other had LE in conjunction with a previously unrecognised Chiari malformation type I. The results of the CT scan were normal.

Patients who suffered PRES and cerebrovascular ischemia developed atrophic brain changes on follow-up MRIs.

**Figure 1:**
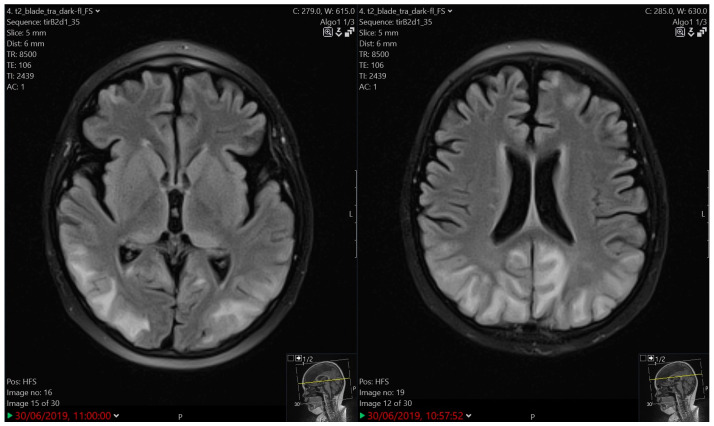
PRES. An MRI finding of subcortical oedema of white matter in occipital and parietal lobes and the posterior segments of the temporal lobes. Smaller oedemas are also observed in the frontal regions.

**Figure 2:**
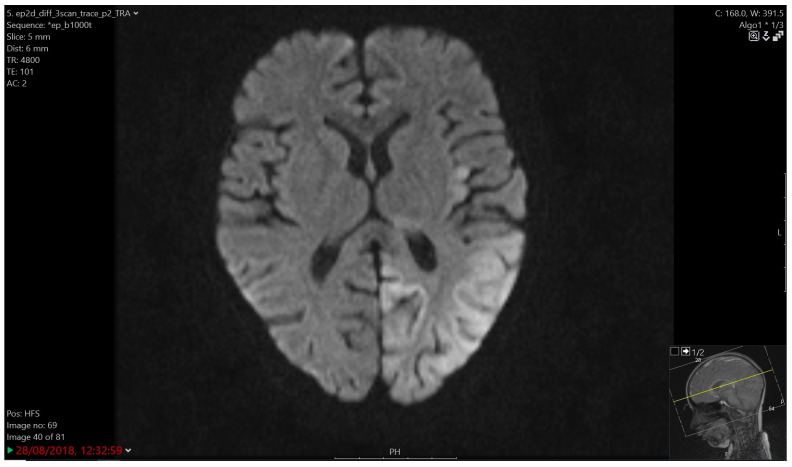
Cerebrovascular ischemia. Ischemia in the supply area of the posterior cerebral artery on the left, including the posterolateral part of the thalamus, the cortex of the occipital, temporal and parietal lobes and the insular area.

**Figure 3:**
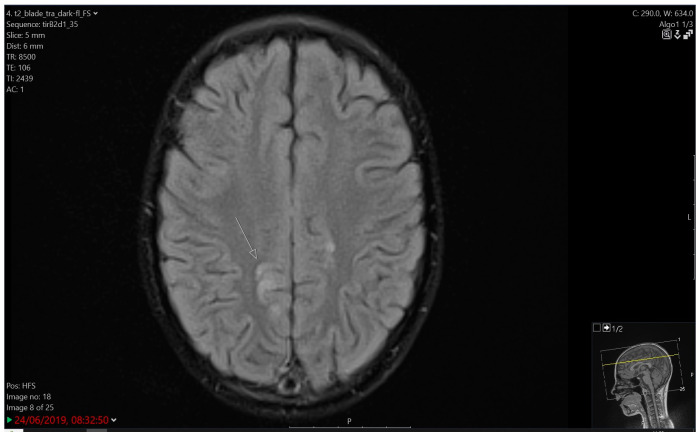
Cortical oedema correlated with the toxic effect of chemotherapy. T2/FLAIR zone of high signal intensity in the posterior segment of the cingulate gyrus on both sides, more pronounced on the right.

**Figure 4:**
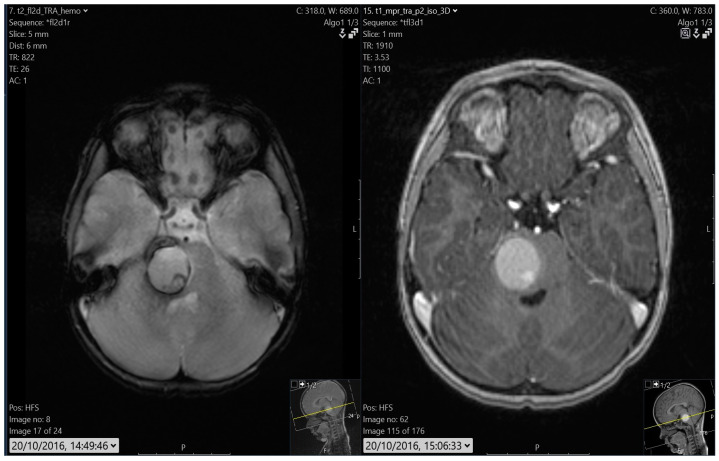
A pontine cavernoma.

The brain imaging findings for all eleven patients are summarised in Table 1, with key examples provided in the following section (Figure 1, Figure 2, Figure 3 and Figure 4).

### 3.5. Treatment and Outcome

Half of the patients (54.5%) received prolonged antiepileptic therapy lasting one to five years. Follow-up EEGs revealed abnormal changes in only one patient; otherwise, complete neurological resolution was observed in all other cases. Chemotherapy modifications were deemed necessary after two incidents. In one case, the final Ara-C block and one administration of intrathecal MTX in Protocol II were omitted, while in another case, the intrathecal therapy was modified.

## 4. Discussion

The incidence of central neurotoxicity in children treated for ALL ranges from 8% to 10% in published larger retrospective studies [26,27,28,29,30]. If children with leukemic CNS infiltration are included in cohorts, the occurrence reaches 17.4% [9,31,32,33]. This high-frequency rate refers to the results of a survey conducted on the population of Mexican children since recent publications have recognised Hispanic ethnicity as a risk factor for neurological adverse events [34,35]. All our patients are Caucasians of European origin; therefore, an incidence rate of almost 20% is double that reported in the literature.

In studies that included more than a thousand children, adolescents and young adults treated for ALL with intensive pediatric protocols, age greater than ten (or six) years was an independent risk factor for the development of neurotoxicity [31,32,33]. Therefore, a higher incidence of central neurological complications in our slightly older patient population might be an expected result. On the other hand, in series with fewer patients [26,27,28], the median age of ALL patients who developed central neurotoxic complications was lower (4.0–5.75 years), most likely related to sample size and a peak ALL incidence in toddlers and preschool children.

ALL is slightly more common in boys [36]. However, central neurotoxicity occurs more often in girls. Female sex in this context is an independent risk factor in a multivariate analysis of more than 3000 patients from the United Kingdom [32]. The female sex prevails as the risk factor in the published large series from Argentina, where children are treated with the same protocol as in Croatia [9]. In contrast, in two studies conducted by Cruz-Chavez and Anastasopoulou, the sex distribution in children affected by CNS toxicity was approximately equal. The female and male gender was equally represented in the total cohort of our patients treated for ALL. Nevertheless, the majority of our patients manifesting neurotoxicity were girls, although this observation did not reach statistical significance.

B-immunophenotype ALL represents 80–85% of childhood ALL [37]. Higher initial leukocyte count, T- immunophenotype and high-risk cytogenetics are related to leukemic infiltration of the CNS [38,39,40]. Chemotherapy used for the T-ALL has the potential risk for neurological complications (higher doses of systemic MTX, prophylactic CNS radiation in certain situations). Still, the T-immunophenotype per se is listed as one of the risk factors for central neurotoxicity [31,32]. Among our patients, only one with T-ALL exhibited central neurotoxicity, contrary to the above-mentioned literature data.

Our patients manifesting central neurotoxicity were initially stratified mainly into a high-risk group and received more intensive chemotherapy. Thus, adverse events, including CNS toxicity, were, not surprisingly, more frequent. Our study regarding the correlation between chemotherapy intensity and the occurrence of adverse central neurological events has, therefore, been consistent with published data [31,32,33]. Neurotoxicity most commonly appears in the most intensive treatment phase, induction and re-induction, regardless of the risk group. However, it is also expected during the consolidation phase of the high-risk disease. Neurological incidents occurred within the first 6 to 8 months after starting the treatment, which is in correlation with previous reports [30,31,41]. Only one patient developed status epilepticus almost two years after diagnosis and one year after CRT. Most adverse events occurred within 21 days of intrathecal MTX application. Other authors have observed a similar timeframe [27,34,42,43,44], which correlates with the well-known MTX-related neurological toxicity.

In the ALL IC BFM 2009 Protocol administered at our institution, three cytostatic agents with possible CNS toxicity are distinguished: MTX, Ara-C and asparaginase.

The mechanism of neurotoxicity of MTX is the most studied so far. MTX neurotoxicity is recorded in 3–7% of children treated for ALL [9,41] and includes aseptic meningitis or chemical arachnoiditis, ‘stroke-like syndrome’ and leukoencephalopathy [42,43,45]. Leukoencephalopathy often appears after combining treatment with high-dose MTX, intrathecal MTX and CRT [16,46,47,48]. Significant chronic complications of MTX therapy include learning disabilities and intellectual decline [49,50,51]. MTX may have a direct cytotoxic effect in the CNS, primarily on astrocytes, which is difficult to verify in practice and has given contradictory results in preclinical trials [52,53,54,55]. This drug also affects several metabolic CNS pathways [37], including nucleic acids synthesis, methylation and metabolism of specific amino acids and cofactors (homocysteine, methionine, S-adenosylmethionine, S-adenosylhomocysteine and biopterin). Nevertheless, genetic markers associated with an increased susceptibility to side effects of MTX administration have not been undoubtedly identified or incorporated into daily practice so far [56].

Unlike MTX, Ara-C and asparaginase neurotoxicity mechanisms are less known. In humans, Ara-C can cause damage in the cerebellar cortex and loss of Purkinje cells [57,58], leading to clinical cerebellar symptoms: dysarthria, nystagmus and ataxic gait [14]. L-asparaginase is associated with cerebrovascular complications (bleeding or thrombosis) caused by an imbalance of anticoagulant and procoagulant factors. During the metabolic breakdown of asparaginase, aspartic acid and ammonia develop, which can be associated with encephalopathy [59,60,61] but in the context of a previous liver lesion [62].

In pediatric oncology centres worldwide, similar to in our department, invasive diagnostic and therapeutic procedures are performed under general anaesthesia. Therefore, the possible neurotoxicity of the anaesthetic agents should be considered. The most frequently used anaesthetics at our centre are fentanyl, propofol and sevoflurane gas. Animal research has shown that sevoflurane alone can lead to neuroapoptosis, the effect enhanced by propofol [63]. However, extensive, well-designed studies (GAS, MASK, PANDA) did not prove that a single, short exposure to general anaesthesia in children is associated with impairment in neurological development [64,65,66]. The role of repeated exposures to general anaesthesia in neurotoxicity remains open, especially with the simultaneous administration of anaesthetic and cytostatic agents. St. Jude study analysed 212 survivors of childhood ALL and detected significant late neurocognitive impairments and imaging changes in the corpus callosum, most likely associated with higher cumulative doses of flurane and propofol, independent of the chemotherapy-related damage [67]. 

Some reports denote corticosteroids and vincristine, in addition to MTX, Ara-C and asparaginase, as potentially neurotoxic drugs. Corticosteroids mostly exhibit side effects on organ systems other than CNS, although psychiatric side effects and pseudotumor cerebri are their well-known adverse events. Vincristine causes peripheral neurotoxicity and a syndrome of inappropriate ADH secretion (SIADH) and was, therefore, also not in the focus of our attention.

No prior neurological morbidity was established in our cohort. However, the concern might be an incomplete initial neurological work-up. The contemporary protocol recommends performing a brain CT scan to assess potential leukemic infiltration of the CNS, apart from cytological analysis of the CSF, as part of the initial diagnostic algorithm. Implementing this diagnostic approach depends on the patient’s condition at diagnosis and requires additional staff engagement. Nevertheless, we perform an initial neurological examination with EEG and brain MRI whenever the patient’s condition and the resources allow us.

Seizures were the most frequently reported adverse neurological events. Seizures can arise from various causes and are identified through a comprehensive neurological examination, laboratory tests, EEG and imaging studies. Understanding the underlying cause is essential for providing appropriate acute care and planning further treatment, rehabilitation and follow-up.

However, situations when the exact cause remains undetermined are not so rare in daily clinical practice. The most common EEG finding among our patients with seizures was slower activity over specific regions and focal changes prone to generalisation. Almost half of the seizures were generalised tonic-clonic. Other authors have also registered generalised semiology in this clinical context as the most frequent one [41]. Neuroimaging, most often an MRI, was performed after each seizure event, with leukoencephalopathy and PRES being the most common findings.

Leukoencephalopathy encompasses radiological changes in white matter, most often periventricular and centrum semiovale, indicating cytotoxic oedema [68]. The symptoms include headache; quantitative, qualitative or both changes of consciousness; seizures; dementia; focal neurological deficits; and speech and vision disorders [34,42,43,45]. Acute manifestations are usually transient. Restricted diffusion on MRI diffusion-weighted images is a reliable early sign of acute MTX encephalopathy [44,68]. It is possible that MRI changes gradually disappear or even resolve completely [42,45,69]. However, subacute and chronic forms of MTX-induced leukoencephalopathy have a more insidious onset and can have severe and permanent sequelae [45]. Five of our patients had MRI changes corresponding to leukoencephalopathy, manifesting itself most frequently with seizures.

PRES is an acute or subacute reversible event that includes specific clinical manifestations (headache, seizures, visual disturbance and mental changes) associated with characteristic findings in the white matter of the cerebrum, most often posteriorly. It is related to the use of certain drugs (immunosuppressants, cytotoxic agents) and other risk factors such as elevated blood pressure and electrolyte imbalance [70]. In one of our patients with PRES, we registered both arterial hypertension and electrolyte imbalance and arterial hypertension in another.

One of our patients with seizures developed ischemic MRI changes in the territory of the left posterior cerebral artery and subsequent development of right-sided hemiparesis.

Another patient with seizures was diagnosed with a brain stem cavernoma as a consequence of CRT. Cavernomas are vascular lesions with dilated thin capillary spaces without brain parenchyma between them. They are associated with irradiation therapy in childhood. They can develop from one to 26 years after the radiation [71].

An additional two patients presented with isolated seizures, accompanied by normal laboratory tests and unremarkable EEG and MRI findings.

The presentation of central neurological adverse events differs in resource-limited and developed countries. In the latter, PRES, cerebrovascular incidents and isolated seizures are most often reported [9,27,31]. Moreover, a decreased level of consciousness, leukoencephalopathy, cerebral venous sinus thrombosis, aseptic meningitis (chemical arachnoiditis) and infections are also possible [28,30,31,37]. In resource-limited countries, CNS infections, bacteremia and sepsis, especially in periods of neutropenia, commonly cause central neurological adverse events [29]. Three of our patients developed seizures during an episode of febrile neutropenia with no isolated microbial agent from blood cultures. In these situations, broad-spectrum antibiotics are the standard of care, and carbapenems can be associated with seizures, further complicating the clinical interpretation [72]. The pattern of central neurological toxicity in our centre is similar to that of developed countries, where leukoencephalopathy, PRES and cerebrovascular accidents predominate.

Considering the severity of these adverse events, they were mostly characterised as moderate due to new-onset, repeated or long-lasting (more than 30 min) seizures, decreased capability of daily self-care and the need for hospital or intensive care unit (ICU) admission. One-quarter were assigned as grade IV, or life-threatening, requiring mechanical ventilatory support.

Our patients successfully recovered from the acute incident, underscoring the crucial role of a multidisciplinary approach in managing the complexities of pediatric oncological treatment. In most cases, pediatric neurologists prescribed antiepileptic therapy, and a child with brainstem cavernoma underwent two neurosurgical procedures. More than half of our patients received prolonged antiepileptic therapy with frequent neurological follow-up, which undoubtedly influenced their quality of life and outcome.

According to the published data and taking into account the risk of relapse, re-exposure to the same antileukemic agents is justified and does not necessarily lead to a new neurological incident [42,45,73]. The chemotherapy protocol continued unchanged in all but two patients whose therapy was modified. No new neurological adverse events occurred, yet with close interdisciplinary collaboration. Regular neurological follow-ups were scheduled for all affected patients. However, due to limited resources, no essential psychological assessments were performed.

## 5. Conclusions

Our observations indicate that relevant risk factors for a central adverse neurological event during childhood ALL treatment include early school age, female gender and recent intrathecal MTX exposure. However, a statistically significant relation was determined solely for high-risk disease. Further research is necessary to explore the potential neurological effects of other medications, particularly those used for sedation and general anaesthesia.

The most common clinical manifestation of acute neurotoxicity is generalised cerebral seizure with different underlying causes—in our cohort, leukoencephalopathy, PRES, cerebrovascular incidents and isolated seizures. The incidents did not require significant chemotherapy modification or termination.

Detailed initial neurological and radiological work-up, as well as structured long-term follow-up, including psychological assessment, are of utmost importance in the management of acute neurotoxicity in children with ALL to achieve optimal treatment outcomes.

## Figures and Tables

**Table 1:** Clinical manifestations of acute neurologic events, diagnostic reports and outcomes.

Patient	Age	Sex	ALL Lineage	Risk	Treatment Phase (AE)	Clinical Presentation (AE)	Duration (AE)	EEG (AE)	MRI (AE)	Grading (CTCAE v. 5.0)	Neurological Outcome
1	8	F	B	IR	Protocol II	Tonic seizure	3 min	Regular	Normal	2	CR
2	8	F	B	HR	Protocol IB	Tonic-clonic status epilepticus	30 min	Lower voltage	Discrete atrophic changes	3	2 years of AET
3	7.5	M	B	HR	Protocol II	Tonic-clonic seizure	5 days	Very slow background activity	PRES	3	1 year of AET, atrophic MRI brain changes
4	7.5	F	T	HR	Protocol IA	Atonic seizure, Subsequent right-sided hemiparesis	2 days	Slower activity, high voltage slow waves left	Cerebro- vascular ischemia	3	Ongoing AET, atrophic MRI brain changes
5	3.5	F	B	HR	Protocol IA	Tonic status epilepticus	10 min	Slower activity bilaterally	PRES	4	CR, atrophic MRI brain changes
6	5	F	B	HR	Consolidation HR-1 block	Tonic-clonic seizure	10 min	Regular	Normal	3	CR
7	7.5	F	B	IR	Protocol II	Tonic-clonic seizure	30 s	Focal changes over FCT bilaterally with generalisation	LE	3	5 years of AET; CTX modification
8A	6	F	B	IR	Protocol II	Tonic seizure	1 min	Slower activity CTP right	LE	3	Selective mutism
8B	10		relapse	N/A	Protocol II-IDA	Complex partial seizure with generalisation; status epilepticus	30 min	Focal changes left FCTO	LE, cortical oedema	4	2 years of AET; ITT modification
9	3	M	B	HR	Maintenance	Complex partial seizure with generalisation; status epilepticus	2 h	Focal changes left	LE, pontine cavernoma, mineralising angiopathy	4	Two neurosurgical procedures; 5 years of AET
10	6	F	B	SR	Protocol IB	Reduced level of consciousness	5 min	Focal changes right	Not performed. Brain CT normal	3	CR
11	4.5	M	B	SR	Protocol II	Headache, vomiting	unknown	Not performed	LE, Chiari malformation type I	2	CR

Abbreviations: ALL—acute lymphoblastic leukemia; AE—adverse event; EEG—electroencephalogram; MRI—magnetic resonance imaging; CTCAE—common terminology criteria for adverse events; M—male; F—female; HR—high risk; IR—intermediate risk; SR—standard risk; FCT—fronto-centro-temporal; CTP—centro-temporo-parietal; FCTO—fronto-centro-temporo-occipital; LE—leukoencephalopathy; PRES—posterior reversible encephalopathy syndrome; AET—antiepileptic therapy; CR—complete resolution; CTX—chemotherapy; ITT—intrathecal therapy.

## Data Availability

The data presented in this study are available on request from the corresponding author in order to preserve patients’ anonymity.
